# Machine-learned and codified synthesis parameters of oxide materials

**DOI:** 10.1038/sdata.2017.127

**Published:** 2017-09-12

**Authors:** Edward Kim, Kevin Huang, Alex Tomala, Sara Matthews, Emma Strubell, Adam Saunders, Andrew McCallum, Elsa Olivetti

**Affiliations:** 1 Massachusetts Institute of Technology, Cambridge, Massachusetts 02139, USA; 2 University of Massachusetts Amherst, Amherst, Massachusetts 01003, USA

**Keywords:** Computational methods, Design, synthesis and processing, Materials science

## Abstract

Predictive materials design has rapidly accelerated in recent years with the advent of large-scale resources, such as materials structure and property databases generated by *ab initio* computations. In the absence of analogous *ab initio* frameworks for materials synthesis, high-throughput and machine learning techniques have recently been harnessed to generate synthesis strategies for select materials of interest. Still, a community-accessible, autonomously-compiled synthesis planning resource which spans across materials systems has not yet been developed. In this work, we present a collection of aggregated synthesis parameters computed using the text contained within over 640,000 journal articles using state-of-the-art natural language processing and machine learning techniques. We provide a dataset of synthesis parameters, compiled autonomously across 30 different oxide systems, in a format optimized for planning novel syntheses of materials.

## Background & Summary

Materials Genome Initiative efforts have led to the proliferation of open-access materials properties databases, resulting in the rapid acceleration of materials discovery and design^1–6^. Given these advances in screening for novel compounds, the realization of comprehensive frameworks for predicting novel synthesis routes is now a primary bottleneck in materials design^7^. Recent high-throughput and data-driven explorations of materials syntheses have focused on optimizing a particular material system of interest^8,9^. Yet, the general landscape of synthesizable materials^10,11^ spanning across material systems remains largely unexplored. To further encourage rapid and open synthesis discovery in the materials science community, we present here a dataset which collates key synthesis parameters aggregated by chemical composition (e.g., BiFeO_3_) across 30 commonly-reported oxide systems.

Many of the largest-volume databases consist primarily of data which are computed *ab initio* (e.g., using density functional theory)^1,12^. There are, however, ongoing efforts which make use of human-collected information extending beyond what can be computed from first principles: Ghadbeigi *et al.*^4^ provide human-retrieved performance indicators for Li-ion battery electrode materials, extracted from ~200 articles, and Raccuglia *et al.*^9^ apply a similar human-data-retrieval technique to lab notebooks to compile ~4,000 reaction conditions for training machine-learned syntheses of vanadium selenite crystals. Additionally, high-throughput experimental syntheses are capable of producing vast combinatorial materials ‘libraries’ for the purposes of materials screening^8,13,14^. These approaches lay the groundwork towards a broader approach using automated data collection techniques.

To accelerate the materials science community towards the goal of rapidly hypothesizing viable synthesis routes, a method for programmatically querying the body of existing syntheses is necessary. Such a resource may serve as a starting point for literature review, or an initial survey of ‘common’ and ‘outlier’ synthesis parameters, or as supplementary input data for other large-scale text mining studies on materials science literature. Indeed, approaches to high-throughput synthesis screening have seen recent success in organic chemistry^15–22^, since organic reaction data is well-tabulated in machine readable formats^23^.

**Figure 1:**
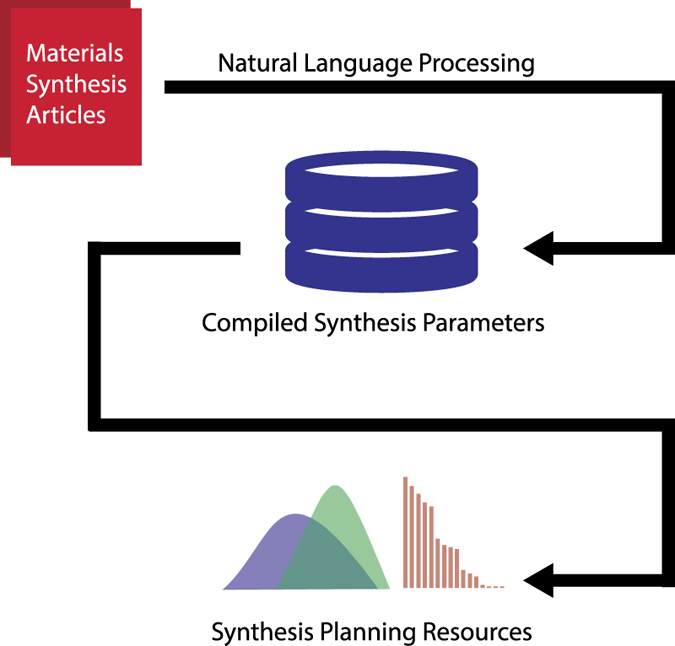
Schematic overview of text extraction and database construction. Each colored object represents a high-level step in the automated workflow for retrieving journal articles and processing text into codified synthesis parameters. Materials synthesis articles are fed into a NLP pipeline, which computes a machine-readable database of synthesis parameters across numerous materials systems. These parameters can then be queried to produce synthesis planning resources, including, empirical distributions of real-valued parameters and ranked lists of keywords.

In this work, we provide a set of tabulated and collated synthesis parameters across 30 oxide systems commonly reported in the literature. This data is retrieved by first training machine learning (ML) and natural language processing (NLP) algorithms using a broad collection of over 640,000 materials synthesis journal articles. These trained algorithms are then used to parse a subset of 76,000 articles discussing the syntheses of our selected oxide materials. Figure 1 provides a schematic overview of the methods used for transforming human-readable articles into machine-readable synthesis parameters and synthesis planning resources. No direct human intervention is necessary in this methodology: Our automated text processing approach downloads articles, extracts key synthesis information, codifies this information into a database, and then aggregates the data by material system.

## Methods

### Article retrieval

Using the CrossRef search Application Programming Interface (API)^24^, journal articles are programmatically queried and downloaded using additional API routes, approved individually by each publisher we access. These articles are downloaded in HTML and PDF formats, and we convert these articles to plain text for further text extraction and processing. PDF articles are converted using the open source *watr-works* Scala program.

### Article section relevance

In order to determine which paragraphs contain materials synthesis information, we have manually applied binary labels to thousands of paragraphs from approximately 100 different journal articles, with positive samples representing materials synthesis paragraphs and negative samples representing all other paragraphs. We use this data to train a binary logistic regression classifier, implemented in *scikit-learn*^25^.

Each paragraph in an article is represented by binary counts of frequently occurring words (commonly referred to as a ‘bag of words’ vector), and this is concatenated with a vector of simple binary heuristics (e.g., if the section title is ‘Experimental’ or ‘Methods’). A logistic regression classifier then applies categorical labels to the paragraphs, with a label of 1 indicating a synthesis paragraph and 0 representing a paragraph unrelated to synthesis. We find an overall F1 score of **96%** using this method, where the F1 score is computed from binary precision (true positives/all positive guesses) and recall (true positives/all positive samples). This score, which emphasizes the ability of a classifier to identify true positive samples, is used since most paragraphs in an article do not describe the synthesis of a material (and so synthesis paragraphs are the rarer category).

$$F1=2\times \frac{\mathrm{precision}\times \mathrm{recall}}{\mathrm{precision}+\mathrm{recall}}$$

### Text extraction

**Figure 2:**
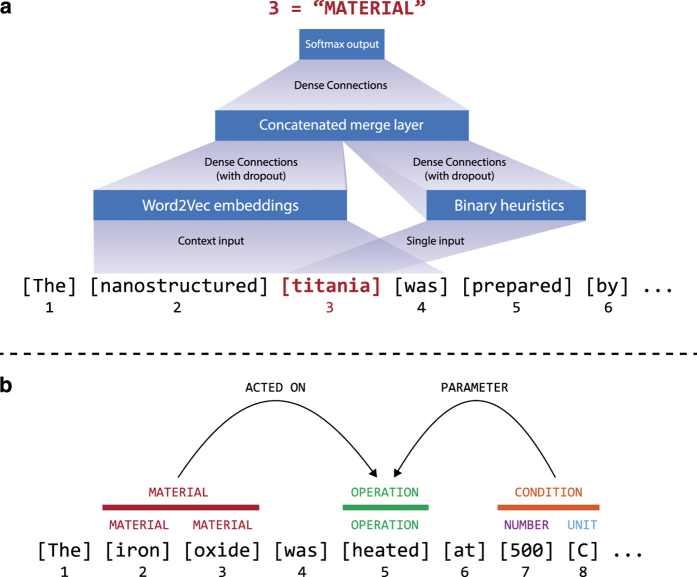
Neural-network and parse-based synthesis parameter extraction. (**a**) A hierarchical neural network assigns labels (e.g., ‘MATERIAL’) to words one-at-a-time by converting words to embedding and heuristic vector representations, and outputting to a classifier. The embeddings of a five-word window are considered for each prediction. Each layer is densely connected, with the hidden layer concatenating each of the two input layers. The final layer is a *softmax* (classifier) computed over each possible word category. (**b**) A grammatical parse of a sentence is used to resolve word-level labels (below colored bars) into sequential word-chunk-level labels (above colored bars), followed by resolution into word-chunk relations (curved arcs).

A schematic overview of the text extraction procedure is provided in Fig. 2. In Fig. 2a, word-level labels are applied using a neural network which predicts the category of each word (e.g., material, amount, number, irrelevant word), using a mixture of word embedding vectors from nearby words and domain-knowledge-driven heuristics. These heuristics include known word matches (e.g., ‘calcine’) and outputs of existing databases and models^18,26,27^ which we have incorporated into our framework. A full description of the word labels is provided in Table 1.

In order to predict word-level labels, a *transfer learning* setup is used^28^, in which we first learn, in an unsupervised manner, a feature mapping function for words using unlabeled data. Then, we use these learned features to create context-sensitive word inputs during supervised training on a smaller set of data with high-accuracy labels applied by humans.

First, the Word2Vec algorithm is pre-trained on 640,000 unlabeled full-text materials synthesis articles in order to learn accurate vector representations for domain-specific words (e.g., anneal), which do not appear frequently in English-language documents^29^. This process yields a transformation function which accepts a plain-text word and outputs a dense, real-valued, fixed-length vector. These word vectors are used alongside the binary heuristic vectors to produce the fully-realized inputs for the neural network.

Following this pre-training, a *baseline* neural network is trained. A set of word labels is computed on this same set of 640,000 articles using only the binary heuristic rules (without the neural network, using simple ‘if…then’ logic). The *baseline* neural network is then trained to mimic the output of these heuristic rules using the embedding vectors as additional supporting data features. The neural network achieves a categorical accuracy of >**99%** and an F1 score of >**99%** on the task of replicating the heuristic rule word labels. This *baseline* version of the neural network achieves a categorical accuracy of **78%** and an F1 score of **66%** on a *test set* constructed from 10 human-labelled articles (~1,700 words). The *baseline* neural network thus serves as a lower-bound accuracy benchmark which represents the effectiveness of a database lookup strategy for categorizing words, using deterministic rules including matches to known chemical formulas (e.g., ‘Fe_2_O_3_’) or known verbs (e.g., ‘sinter’).

Separately, a *human-trained* neural network learns from annotated data labels which have been applied by materials scientists: A training set of 20 articles (~5,200 words) with human-applied word labels is used to learn the weights of the neural network. These human-applied word labels are marked while reading through the synthesis sections of journal articles, and thus emphasize scientific and linguistic context rather than a strict adherence to deterministic rules and database lookups. This *human-trained* network indeed achieves higher accuracies compared to the *baseline*: the categorical accuracy of this neural network classifier, as measured against the same *test set*, is **86%** and its F1 score is **81%**. This is comparable to the performance achieved by the recent *ChemDataExtractor* model on a similar task^18^, which is trained to extract relevant text from chemistry articles.

Figure 2b shows the process of interpreting higher-level relations in the text, including resolving multiple sequential words into a single ‘chunked’ entity and relating these chunks to each other. This process is done by applying heuristic rules to the outputs of a grammatical parser^27^, where relations between chunks are computed from parse tree dependencies, with word-order proximity used as a fallback measure^30^. To illustrate via the example in Fig. 2b, ‘500 C’ is grammatically dependent on ‘heated,’ and so a relation is assigned between them.

The extracted synthesis parameters from these articles are then filtered by material systems of interest, and aggregated for each material. Histograms and ranked lists of keywords are computed by counting occurrences within documents, and all histograms are normalized to integrate to unity. In this paper, we present a dataset extracted from a sample of 76,000 articles which discuss the syntheses of 30 oxide systems of interest. Beyond this, we also provide a more extensive and continuously-updated dataset at [www.synthesisproject.org].

### Code availability

The code used to compute and analyze the data is available at [www.github.com/olivettigroup/sdata-data-plots/]. Additionally, the compiled Word2Vec embedding vector model is available at [www.github.com/olivettigroup/materials-word-embeddings/]. The underlying machine learning libraries used in this project are all open-source: *Tensorflow*^31^, *SpaCy*^27^, and *scikit-learn*^25^.

## Data Records

The data are provided as a single JSON file, available at [www.synthesisproject.org] and through figshare (Data Citation 1). Each record, corresponding to data for a single material system, is represented as a JSON object in a top-level list. The details of the data format are given in Table 2.

**Figure 3:**
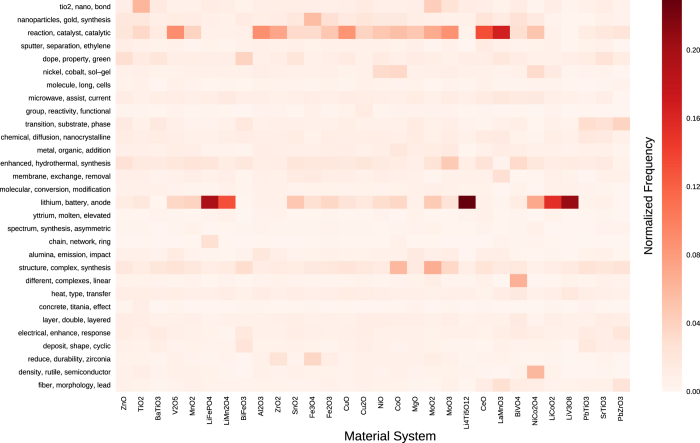
Topic and synthesis target distributions within the database. Heatmap showing a sample of topic distributions plotted against material systems of interest. Topics are computed from training a Latent Dirichlet Allocation model on 640,000 journal articles, and are labelled by their top-ranked keywords^32^. Values of the heatmap represent column-normalized counts across all articles within a material system.

Metadata for each material system is provided in the JSON dataset, including topic distributions computed with Latent Dirichlet Allocation^32^. These topic distributions are visualized as a heatmap in Fig. 3, and demonstrate correlations between material chemistries and device applications, experimental apparatuses, and product morphologies.

**Figure 4:**
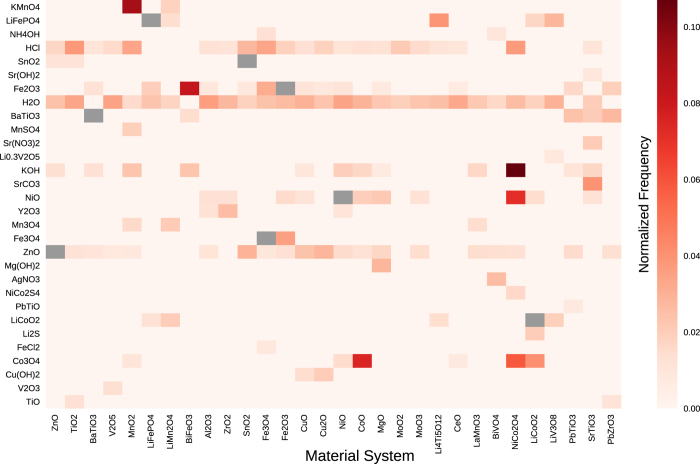
Top occurring material mentions per target material system. Heatmap showing a sample of co-occurring mentions of materials within synthesis routes for material systems of interest. Values of the heatmap represent column-normalized counts across all articles within a material system. Counts of self-mentioning co-occurrences (e.g., ZnO mentioned in papers synthesizing ZnO) are fixed to zero prior to column normalization and plotted in grey.

Numerical synthesis parameters (e.g., calcination temperatures) for each material system are provided as kernel density estimates, computed across all journal articles discussing the synthesis of a given material. Such a format allows for rapid visualizations to aid high-level synthesis planning: for example, Fig. 4 shows co-occurrences between materials systems and mentions of other materials extracted from synthesis sections of articles.

## Technical Validation

**Figure 5:**
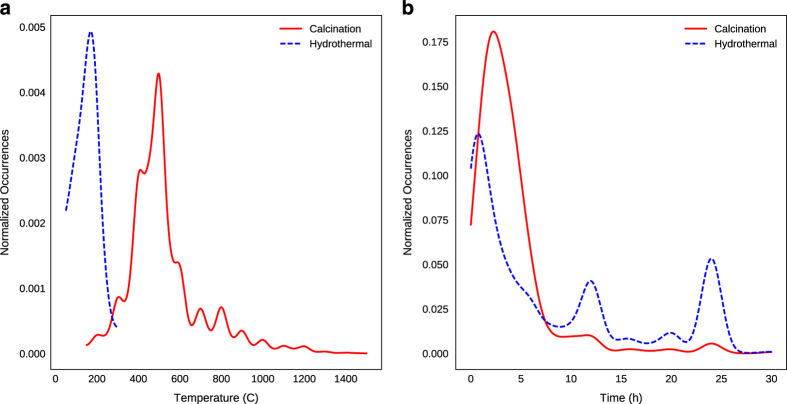
Temperature and time distributions for titania. (**a**) Calcination and hydrothermal temperature kernel density estimate for titania, normalized to unit area. (**b**) Calcination and hydrothermal time kernel density estimate for titania, normalized to unit area. All density estimates are computed using Gaussian kernels computed from counts of temperatures and times extracted from synthesis sections of journal articles.

For scientific validation, we briefly compare the aggregated data in our provided dataset to known results. As an example, Fig. 5a displays frequent usages of temperatures near the anatase-rutile phase boundary for titania^33^. This data thus agrees with the intuitive reasoning that such temperatures are used to either crystallize an anatase-phase product, or convert to a rutile-phase product^34^. We also observe additional patterns which agree with intuitive expectations: Fig. 5a shows that hydrothermal reactions are confined to a narrow temperature range, peaking between 100–200 C, and Fig. 5b confirms that hydrothermal reactions more typically occur for long periods of time compared to calcination.

**Figure 6:**
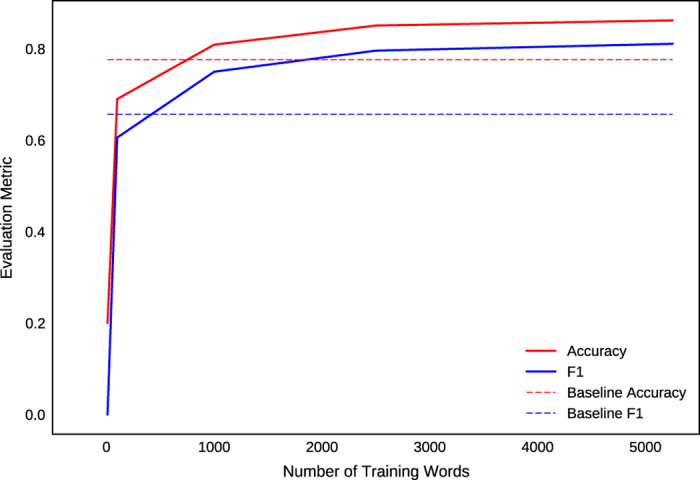
Learning curve for neural-network word classifier. The baseline accuracy and F1 score are plotted as horizontal lines, computed from the *baseline* neural network on the maximum number of training words. The solid curves are computed from the *human-trained* neural network, showing accuracy and F1 score as a function of training data volume.

Besides validating the accuracy of the text extraction and word-labelling methods (as discussed in the Methods), we reiterate here the accuracy of the parsing algorithm used, which is reported as **91.85%** (ref. 27). We use this parsing algorithm as a dependency to resolve higher-level relations in our extracted text data (e.g., relating ‘500 C’ to ‘heated’ in Fig. 2b). We also report a training curve in Fig. 6, to demonstrate that several thousand labelled words is indeed a sufficient volume of data for training a neural network word classifier in the materials science domain.

## Usage Notes

As this data is provided in the language-agnostic JSON format, no specific technical setup is required as a dependency. The authors have found it useful to load the data into the *Python* programming language, especially for downstream integration with data from the Materials Project provided via their *pymatgen* library^35^.

A detailed, web-accessible Python tutorial for loading and analyzing the dataset is available at [https://github.com/olivettigroup/sdata-data-plots/blob/master/SDATA-data-plots.ipynb]. This web tutorial provides the exact Python code used to generate the figures in this article, along with commentary which explains the technical setup.

Empirical histograms provided in this dataset, along with ranked lists of frequent synthesis parameters, serve as useful starting points for literature review and synthesis planning: for example, selecting the most frequent synthesis parameters (e.g., most common reaction temperatures and precursors) would yield a starting point for a viable synthesis route.

Additionally, the topic labels provided in this dataset may prove useful in studies related to metadata and text mining in the materials science literature. As a motivating example, authorship and citation links have been analyzed in biomedical papers to reveal insights related to the impact of papers over time^36^; such analyses could potentially be extended to topic models in materials science.

While this paper details a static ‘snapshot’ of collated synthesis data, a continuously updated and rapidly-expanding dataset is also available via an API at [www.synthesisproject.org].

## Additional Information

**How to cite this article:** Kim, E. *et al.* Machine-learned and codified synthesis parameters of oxide materials. *Sci. Data* 4:170127 doi: 10.1038/sdata.2017.127 (2017).

**Publisher’s note:** Springer Nature remains neutral with regard to jurisdictional claims in published maps and institutional affiliations.

## Supplementary Material



## Figures and Tables

**Table 1:** In-domain word categories and examples.

**Word Label**	**Interpretation**	**Examples**
TARGET	Final synthesized material	TiO_2_, BiFeO_3_
UNSPECIFIED	Generic references to materials	Solution, powder
MATERIAL	Non-target named materials	TiCl_4_, NaOH
OPERATION	Action on a material	Dissolve, sinter
AMOUNT MISC	Unspecified amount	Several, dropwise
AMOUNT UNIT	Amount-type unit	ml, mmol
CONDITION MISC	Unspecified parameter of an action	Slowly, ambient
CONDITION UNIT	Unit for parameter of an action	hours, °C
SYNTHESIS APPARATUS	Experimental equipment	Autoclave, furnace
CHARACTIZATION APPARATUS	Experimental equipment	s.e.m., diffractometer
DESCRIPTOR	Qualitative material morphology	Layered, nanorods
PROPERTY MISC	Qualitative aspect of a material	Denser, brittle
PROPERTY UNIT	Quantitative aspect of a material	MPa, cm^2^
PROPERTY TYPE	Type of aspect of a material	Strength, area
NUMBER	Numerical words	120, five
META	Synthesis route type	Solvothermal, sol-gel
BRAND	Commercial brand	Sigma, Fisher
REF	Citation or reference	[14], 2007
NULL	All other words	The, before
Word-level labels and examples of words belonging to each label. Each word in an article is assigned to exactly one of these labels.		

**Table 2:** Schema of data records.

**Data Description**	**Data Key Label**	**Data Type**
Name of material system	Name	String
Number of papers used to compute synthesis parameters	Num_papers	Integer
Top occurring synthesizing actions used for a material system	Associated_operations	Array of strings
Top co-occurring materials in synthesis sections for a material system	Associated_materials	Array of strings
Topic distribution for a material system	Topics	Object (dictionary) of topic strings and frequency floats
All temperatures reported in syntheses for a material system, aggregated as a kernel density estimate	Temperature_kde	Object (dictionary) of x and y floats
Hydrothermal temperatures and times reported in syntheses for a material system, aggregated as a kernel density estimate	Hydrothermal_kde	Object (dictionary) of x and y floats
Calcination temperatures and times reported in syntheses for a material system, aggregated as a kernel density estimate	Calcine_kde	Object (dictionary) of x and y floats
Overview of formatting for each data record, with each row representing a data record key. For each key, the key label name is provided, along with the data type.		
